# State and Training Effects of Mindfulness Meditation on Brain Networks Reflect Neuronal Mechanisms of Its Antidepressant Effect

**DOI:** 10.1155/2016/9504642

**Published:** 2016-02-21

**Authors:** Chuan-Chih Yang, Alfonso Barrós-Loscertales, Daniel Pinazo, Noelia Ventura-Campos, Viola Borchardt, Juan-Carlos Bustamante, Aina Rodríguez-Pujadas, Paola Fuentes-Claramonte, Raúl Balaguer, César Ávila, Martin Walter

**Affiliations:** ^1^Departamento de Psicología Básica, Clínica y Psicobiología, Universitat Jaume I, 12071 Castellón, Spain; ^2^Clinical Affective Neuroimaging Laboratory, 39120 Magdeburg, Germany; ^3^Department of Behavioral Neurology, Leibniz Institute for Neurobiology, 39118 Magdeburg, Germany; ^4^Departamento de Psicología Evolutiva, Educativa, Social y Metodología, Universitat Jaume I, 12071 Castellón, Spain; ^5^Departamento de Didáctica de la Matemática, Facultad de Magisterio de la Universidad de Valencia, 46022 Valencia, Spain; ^6^Departamento de Psicologia y Sociologia, Universidad de Zaragoza, 50009 Zaragoza, Spain; ^7^Department of Psychiatry and Psychotherapy, Otto-von-Guericke University, 39120 Magdeburg, Germany; ^8^Center for Behavioral Brain Sciences (CBBS), 39120 Magdeburg, Germany; ^9^Department of Psychiatry and Psychotherapy, Eberhard Karls University, 72076 Tuebingen, Germany

## Abstract

The topic of investigating how mindfulness meditation training can have antidepressant effects via plastic changes in both resting state and meditation state brain activity is important in the rapidly emerging field of neuroplasticity. In the present study, we used a longitudinal design investigating resting state fMRI both before and after 40 days of meditation training in 13 novices. After training, we compared differences in network connectivity between rest and meditation using common resting state functional connectivity methods. Interregional methods were paired with local measures such as Regional Homogeneity. As expected, significant differences in functional connectivity both between states (rest versus meditation) and between time points (before versus after training) were observed. During meditation, the internal consistency in the precuneus and the temporoparietal junction increased, while the internal consistency of frontal brain regions decreased. A follow-up analysis of regional connectivity of the dorsal anterior cingulate cortex further revealed reduced connectivity with anterior insula during meditation. After meditation training, reduced resting state functional connectivity between the pregenual anterior cingulate and dorsal medical prefrontal cortex was observed. Most importantly, significantly reduced depression/anxiety scores were observed after training. Hence, these findings suggest that mindfulness meditation might be of therapeutic use by inducing plasticity related network changes altering the neuronal basis of affective disorders such as depression.

## 1. Introduction

Mindfulness meditation has been shown to be therapeutic in emotion regulation [1–5]. Mindfulness practice, inherited from the ancient Buddhist tradition, involves observing thoughts nonjudgmentally in the present moment. Mindfulness-based meditation (MBM) or mindfulness-based intervention (MBI) is increasingly being employed in western psychology to alleviate a variety of mental and physical conditions including obsessive compulsive disorder and anxiety and in the prevention of relapse in depression and drug addiction [6–10]. Despite the fact that recent neuroimaging studies indicate the several positive aspects of mindfulness practice, its neuronal mechanisms are still poorly understood.

A main theme in meditation research is the question if the observed effects are dependent on practice/expertise or on personal characteristics. Usually, studies on meditation compare expert practitioners with novice practitioners in cross-sectional studies [11–13]. Functional studies have employed fMRI block designs to investigate the blood-oxygen level dependent (BOLD) signal changes during meditation [11, 12, 14], as well as exploring resting state fMRI (rs-fMRI) differences between experienced meditators and novices [11, 13, 15]. Crucially, longitudinal studies of the impact of short-term meditation practice on the same novice group have the potential to reflect effects of meditation independently of individual differences, for example, personal interest in meditation or previous meditation experience. Hölzel et al. [4] observed changes in gray matter density in a longitudinal study with 8 weeks of a mindfulness-based stress reduction (MBSR) programme.

Resting state functional connectivities (RSFC) methods provide one approach for exploring how mindfulness meditation alters neural plasticity among brain regions. The interregional dynamics of RSFC specifically afford the advantages of being task independent, rendering reliable estimates of neural circuit functionality corresponding to structural topography [16, 17].

To investigate the impact of mindfulness practice on the neuronal level and its antidepressant effects, alteration in the activation pattern of the Default Mode Network (DMN) is of particular interest and the DMN is thus selected as the target brain network [13]. We know that the DMN involves several brain regions active when the brain is not actively engaged in a cognitively demanding task but rather is in a relaxed state [18]. Consistently, the DMN includes areas at the medial posterior cortex, specifically the posterior cingulate cortex (PCC; areas 23/31), the precuneus, and the medial frontal cortex (MFC, including areas 24/10-m/32), as well as bilateral inferior parietal and posterior temporal areas around the temporoparietal junction area (TPJ) [19].

Recent findings suggested that the DMN has altered resting state functional connectivity between networks when depressed patients were compared to healthy participants [20]. Among these DMN regions, dmPFC was shown to be involved in self-inspection [21] and emotion regulation [22] and demonstrated increased activity in depression [23]. Thus, major depressive disorder (MDD) patients show more neural functional connectivity between the posterior cingulate cortex and the subgenual-cingulate cortex during rest periods compared to healthy individuals [24].

Several regions of anterior cingulate are of importance in depression-related disorders. Pregenual anterior cingulate cortex (pgACC) has been shown to be hypoactivated in MDD [25]. The dorsal anterior cingulate cortex (dACC) is associated with the involvement of cognitive control over attentional resources [26, 27], and it is strongly impaired in MDD. van Tol et al. [28] showed that dACC and pgACC are also structurally affected in MDD, while pgACC was furthermore found to be molecularly affected by altered glutamate concentrations. In contrast to the dACC, the pgACC belongs to an affective subdivision of the ACC [26] and was shown to mediate the increased internal focus present in the ruminative thinking style of depressed patients. Furthermore, the pgACC is specifically impaired in highly anhedonic patients [29].

The neurobiological model of depression [30] states that impaired cognitive control, mediated by regions in the prefrontal cortex (PFC), cooccurs with hyperactivation of the amygdala, which facilitates encoding and retrieval of emotional stimuli via modulation of hippocampal activity. Activity in the medial prefrontal cortex (MPFC), which is associated with internal representations of the self, is consequently increased as well. The suppressed regulatory influence of PFC regions facilitates an undesired recall of negative (mood-congruent) events. The DMN has been shown to support internally oriented and self-referential thoughts and MDD has been associated with both hyperconnectivity within the DMN and hyperconnectivity between frontoparietal control systems and regions of the default network [31]. Furthermore, MDD has been characterized by hypoconnectivity within the frontoparietal network, a set of regions involved in cognitive control of attention and emotion regulation, and hypoconnectivity between frontal systems and parietal regions of the dorsal attention network involved in attending to the external environment [31]. These networks modulate affective and cognitive processes disturbed in depression.

In the cases described here, impairment refers to increase or decrease of an existing functional connection. Alterations reflect changes in operational properties of interregional connections and their local resting state activity. As much as MDD is characterized by hyperactivity in, for example, ventral and subcortical regions, this was discussed to be directly related to hypoactivity and resulting disinhibition from dorsal cortical regions [30]. Likewise, within-network connectivity can be altered in opposite direction compared to between-network connectivity (in terms of functional connectivity) as soon as a hub region is concerned, which connects both to its own functional module and towards regions outside its own functional subnetwork.

Recent neuroimaging publications benefit from the utilization of multiple imaging methods. Resting state fMRI widely used seed-based correlation analysis between regions of interest (ROIs) by investigating interregional functional connectivity to reveal highly consistent patterns of functional connectivity across regions [32]. In this study we selected two ROIs in the ACC, pgACC, and dACC, due to their role in emotion regulation and as portions of the DMN. Earlier studies showed that the pgACC was associated with internal monitoring [33, 34] and Mood States [35]. Likewise, the dACC has been shown to relate to focused attention [26, 27] and recent study from Dickenson and colleagues had found that the dACC was recruited during mindfulness meditation as compared to the mind wandering for the novice practitioner [36]. Both pgACC and dACC are therefore target regions for specific symptoms in depression related to mindfulness and emotional/interoceptive awareness, and we focused our analysis on these two regions with supposedly differential, if not contrary, effects, as previously reported in patient studies and suggested by their involvement in potentially antagonistic networks. The method of seed-based functional connectivity was used to test their resting state behavior both before and after training. Furthermore, independent component analysis (ICA), which neither relies on* a priori* model of brain activity, parcellation into ROIs, nor choice of seed regions but has been widely used to detect resting state networks [37], was chosen as a complementary, data-driven approach. We hypothesized that some regions within DMN would be relevant to emotional regulation and ICA was used for functional parcellation of the DMN. In addition to seed-based functional connectivity method and ICA, which reflect the interregional neuronal properties between distant brain regions, ReHo was used to map the level of regional activity synchronization across the whole brain [38]. Regional Homogeneity (ReHo) measures the similarity of the time series of a given voxel to those of neighbouring voxels [39], which reflects the temporal homogeneity of localized neural dynamics [40–42].

Therefore, our aims were to analyze both the interregional and local features of fMRI signal during resting state (RS) in a longitudinal study of RS activation, as well as comparing RS activation to meditation state (MS) activation. We hypothesized that the training of mindfulness meditation will lead to changes in the following: (1) the interregional functional connectivity of our a priori seed ROIs—pgACC and dACC (by means of seed-based FC); (2) the internal consistency of the DMN (by means of ICA); and (3) the regional synchronization of fMRI time series (by means of ReHo).

## 2. Materials and Methods

### 2.1. Subjects

Thirteen university students (Spanish native speakers) were recruited by university advertisement to participate in 40 days of mindfulness meditation course. Sample demographic characteristics are detailed in Table 1. All study protocols were approved by the Institutional Review Board of the Universitat Jaume I of Castellón and informed consent was obtained from all subjects. Participants were screened for psychiatric or neurological conditions prior to enrollment in the meditation course. No previous meditation experience was reported by the subjects.

### 2.2. Mindfulness Meditation Training

The mindfulness training programmes consisted of 8-week courses, with daily practice at home in sessions of around 45 minutes [43]. The mindfulness-based stress reduction (MBSR) programme [44] as well as acceptance and commitment therapy [45] was used to design the meditation-mindfulness training programme based on self-observation training. The self-observation training programme consisted of eight 1.5-hour sessions over a period of 8 weeks. The first hour of the sessions was devoted to simple physical and breathing exercises during which the participants were instructed to perform Vipassana meditation exercises, by focusing their attention on thoughts that came to their mind without dwelling on any of them. After the exercises, participants meditated in silence, continuing to observe their thoughts without censoring them. The time devoted to meditation without the physical exercises gradually increased over the course. The final half hour was used to talk about the experience and explain the characteristics of meditation and mindfulness in which videos and fables reflecting the most significant aspects of the meditation and self-observation experience were used. Participants were also encouraged to do the meditation exercises at home; they were given a meditation diary in which to record their daily experiences, which was handed in to the course instructor. A quantification of the participants' amount of meditation practice at the end of the course is detailed in Table 1.

### 2.3. Self-Report Measures

Three self-assessment questionnaires were administrated to participants both before and after the training period. We applied the Profile of Mood States (POMS) throughout the study in its abbreviated, Spanish version [46] of the original POMS [47]. It is a 44-item inventory, which measures current mood state by rating statements on a Likert scale (0 to 4). It consists of six subscales: anger, fatigue, tension, depression, vigor, and friendliness. To evaluate changes in depressive symptoms of our sample, we used the Center for Epidemiologic Studies Depression Scale (CES-D) which is a well-validated, 20-item inventory [48]. Subjects were asked to rate statements based on the previous week on a Likert scale (0–3) and scores range from 0 to 60, whereat higher scores indicate higher levels of depression. The State-Trait Anxiety Inventory [49] is a 40-item scale designed to measure the state and trait anxiety based on a Likert scale (1–4), which was also applied. Scores range from 20 to 80 whereat higher scores relate to higher levels of anxiety.

### 2.4. Data Acquisition

In this longitudinal study participants were scanned twice. Before the training of mindfulness meditation, subjects underwent a single resting state (RS) scan (time point 1, TP1). Later on, at day 40 (time point 2, TP2), subjects underwent two scans, RS scan and a scan during which they practiced meditation (MS). During the RS, subjects were instructed to close their eyes, be at the normal relaxing condition without engaging in any specific task or mental activity, and not fall into meditation. During meditation scan, subjects were instructed to close their eyes, openly monitor the surrounding environment by accepting all sensations rise and fall nonjudgmentally, and specifically to be aware of the present moment as they were trained. At time point 2 (TP2), RS scan was measured for 9 minutes followed by continuous MS lasting for 12 minutes. Long time acquisition for both conditions was set to obtain sufficient power for the functional connectivity analyses given the relatively small sample size [37].

### 2.5. MR Sequence Parameters

MR measurements were performed on 1.5 T Siemens AVANTO scanner (Siemens Erlangen, Germany). A structural image was acquired from each subject with a magnetization-prepared rapid gradient-echo (MP-RAGE) sequence (TR = 2200 ms, TE = 3.79 ms, flip angle (FA) = 15°, 160 slices, matrix size = 256 × 256, field-of-view (FOV) = 256 mm × 256 mm, and slice thickness = 1 mm). For both RS and MS, a standard EPI sequence was used (TR = 2300 ms, TE = 55 ms, FA = 90°, FOV = 224 mm × 224 mm, matrix size = 64 × 64, and slice thickness = 4 mm,) with 25 axial slices for whole brain coverage. Finally, an extra gradient field mapping sequence (gre_field_mapping) was acquired followed by each EPI sequence (TR = 487 ms, TE1 = 8 ms, TE2 = 12.76 ms, FA = 65°, FOV = 224 mm × 224 mm, matrix size = 64 × 64, and slice thickness = 4 mm) with 25 slices with the same coverage used in EPI sequence.

### 2.6. Data Preprocessing

B0 inhomogeneity correction was performed to reduce static field inhomogeneity using an EpiUnwarping tool based on FSL (http://surfer.nmr.mgh.harvard.edu/fswiki/epidewarp.fsl) [50]. Preprocessing was performed using Statistical Parametric Mapping (SPM8, http://www.fil.ion.ucl.ac.uk/spm/) and Data Processing Assistant for resting state fMRI (DPARSF v 2.3 [51]). Functional images were slice-time corrected. Motion correction was performed by using a least squares approach and a six-parameter (rigid body) linear transformation. Spatial normalization to MNI space was carried out by using unified segmentation of T1-weighted acquired images, and the extracted normalization parameters from segmentation were applied to normalize the functional volumes for each participant (normalized images were then resampled to 3-mm isotropic cubic voxels). Finally, functional volumes were smoothed by applying a 4 mm FWHM Gaussian kernel. Smoothed volumes were used for ICA and seed-based FC. ReHo analysis was performed on nonsmoothed functional volumes and images were smoothed after the analysis, because smoothing prior to ReHo calculation would increase regional similarity [51].

We conducted additional preprocessing for seed-based FC and ReHo analyses using the DPARSF tool through the following steps: (i) removing a linear trend in the time series and (ii) temporal band-pass filtering (0.01–0.08 Hz) to reduce the effect of low frequency drift and high-frequency noise [52, 53]. For the seed-based FC analysis, several sources of spurious variance were removed from the data through linear regression: six parameters from rigid body correction of head motion, white matter signal, cerebrospinal fluid signal, and the global mean signal [54].

For MS, the first and the last 40 volumes of the functional images were discarded (total 313 volumes were acquired before removing the 80 volumes). The reason for removing the first 40 volumes was to allow the participants to get used to the scanning environment and ensure a reasonable establishment of the meditation state. The reason for removing the last 40 volumes was aiming at avoiding distraction effects at the end of the meditation, yielding a duration similar to the RS. The central 233 (8 min 33 sec) functional volumes of the times series were sufficient for estimation of independent components (ICs) [37].

### 2.7. Seed-Based Functional Connectivity

In addition to a data-driven approach, we performed a hypothesis-driven seed-based FC analysis. Two independent seed ROIs placed in the pregenual anterior cingulate cortex (pgACC) and the dorsal anterior cingulated cortex (dACC) were chosen based on previous reports on their involvement in meditation and affective disorders such as depression [55].

**Figure 1:**
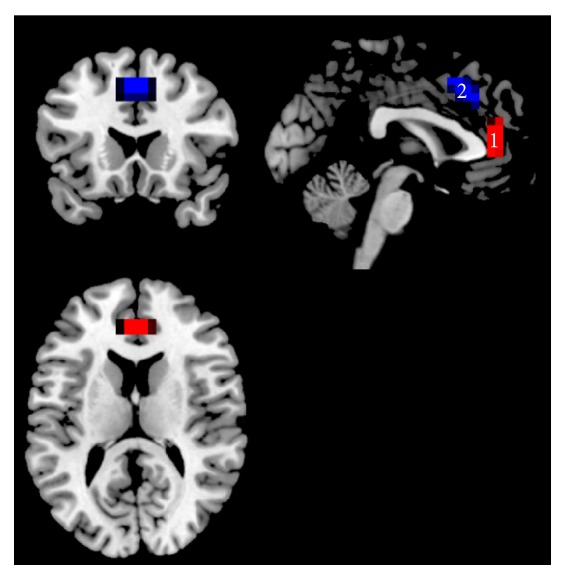
Seed ROIs used for RSFC analysis. ROI1: pregenual ACC (pgACC) as marked in red color. ROI2: dorsal ACC (dACC) as marked in blue color overlaid on the T1 anatomical MNI space.

To examine FC in the pgACC and the dACC in a whole brain voxel-wise analysis, these two seed regions were defined by the Montreal Neurological Institute (MNI) coordinates (*x*, *y*, *z*): 0, 41, and 9 (pgACC) and 0, 27, and 30 (dACC). Both ROIs had a radius of 10 mm resulting in a volume of approximately 4 mL, maximizing the gray matter contribution as described previously [56], as shown in Figure 1. The averaged time course from each seed region was obtained for each subject at both time points and for both conditions (RS, MS) separately. An individual FC map for each seed was generated by calculating the voxel-wise correlation coefficients in the whole brain, which were then converted into *z*-maps by Fisher's *Z* transformation to enhance normality. The *z*-maps of the dACC and pgACC of each individual were entered into second-level paired *t*-test.

### 2.8. Group Independent Component Analysis (Group ICA)

A group independent component analysis was performed using GIFT (v 2.0e, http://mialab.mrn.org/software/gift/) [57] to investigate the following: (a) the training-related RSFC changes and (b) the FC difference between RS and MS. These analyses were performed based on FC analysis from spontaneous BOLD activity from the average fMRI time course of the entire brain.

In the analysis of training-related RSFC changes over time, the longitudinal resting state data were grouped together when conducting group ICA following a previous report [58]. In the analysis of FC differences between RS and MS experimental conditions, a single ICA was performed at the group level for RS and MS conditions separately, in agreement with previous studies [59, 60]. The data of each condition was restricted to twenty-four independent components (ICs) using the MDL criteria and extracted by ICA decomposition using the Infomax algorithm [61]. To determine the reliability of the ICA algorithm, multiple runs of ICA were performed using ICASSO. Each IC consists of a spatial map and an associated time course. The ICs which resembled the DMN most were firstly spatially sorted by using the DMN template built-in in GIFT [57] and then further identified by visual inspection by two different raters for RS and MS separately. Subsequently, individual subject maps were back reconstructed to obtain single-subject results. Finally, the resulting individual IC maps were converted into voxel-wise *z*-score maps representing the degree to which each voxel belongs to the overall ICA component map.

Individual subject *z*-maps were entered into SPM random-effect analyses. The RSFC analysis involved a longitudinal analysis of changes in temporal correlations in connectivity fluctuations before and after training. To compare RS and MS conditions, one paired *t*-test was performed. In the IC decomposition, only DMN was extracted to be compared between time points and conditions. We computed the DMN from the average fMRI time course from the entire network as described before. Furthermore, in the analysis between RS and MS, the DMN comparison was not restricted to a mask of the conjunction between both conditions, since it would involve the restriction of any other regions that may be identified as the DMN in the MS as different from the DMN in the RS, but to the addition of the masks for the DMN identified under RS and MS.

Additionally, exploratory mask restricted analysis was performed but not reported because its contribution was similar to the unrestricted comparison in the target regions of the DMN, although covering differences in other out regions.

### 2.9. Regional Homogeneity (ReHo)

ReHo images of the whole brain for both MS and RS were generated using DPARSF. Voxel-by-voxel Kendall's coefficient of concordance (KCC, [39]) of the time series of a given cluster of 27 neighbouring voxels was calculated [62]. To reduce the global effects of individual variability across participants, the ReHo of each voxel was scaled by the mean value of whole brain ReHo for each participant [20].

A paired *t*-test was performed to identify the effects of meditation on ReHo during resting state activity between TP1 and TP2. A second paired *t*-test was performed to identify the differences in ReHo between the resting states (RS) and meditation state (MS) at TP2. The resulting *t*-value maps of each contrast of interest was displayed after applying a statistical height threshold of *p* < 0.001 for each voxel and a corrected cluster threshold at *p* < 0.05, as determined by a Monte Carlo simulation (see AlphaSim in AFNI http://afni.nimh.nih.gov/pub/dist/doc/manual/AlphaSim.pdf).

## 3. Results

### 3.1. Behavioral Self-Report Measures

A paired *t*-test analysis of the total scores of CES-D indicated a significant reduction of depression scores after meditation training (*t*(12) = 4.43; *p* < 0.001). Before meditation training, the mean CES-D score of the sample was 16.23 ± 9.54, which slightly surpassed the cutoff score for depression (a CES-D above 16 indicates depression; see [63]). After training, the mean CES-D score was reduced to 9 ± 6.20.

Pre-post comparisons of the STAI measures revealed a significant reduction in trait anxiety (*t*(12) = 2.76; *p* < 0.01), but not in state anxiety scores. Neither the sum score of the POMS nor its sub-scores showed a significant change (all *p* < 0.05, controlled for multiple comparisons). However, the tension subscore did show a change (*t*(12) = 1.883, *p* < 0.05, uncorrected); see Table 2.

As an exploratory step, the changes in CES-D scores were correlated with the changes in connectivity of the FC components, but the results were not significant for dACC (*r* = .122, *p* > 0.1) and pgACC (*r* = −.064, *p* > 0.1).

### 3.2. Seed-Based Functional Connectivity

#### 3.2.1. Comparison of FC Differences between RS and MS

**Figure 2:**
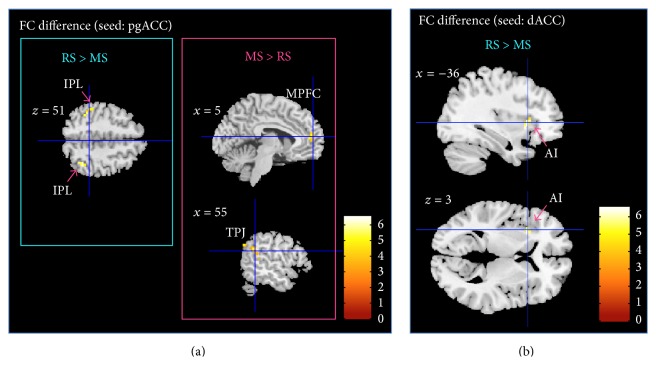
(a) Paired comparison of seed-based FC maps (seed = pgACC) between rest and meditation at TP2 (*p* < 0.05, FWE-corrected). Bar plot represents the *t*-values. White labels indicate the coordinate of each slice in the MNI frame of reference (*x*, *z*). (b) Paired comparison of seed-based FC maps (seed = dACC) between rest and meditation at TP2. FC decreases between dorsal ACC and left AI during meditation as compared to rest (*p* < 0.05, FWE-corrected). Bar plot represents the *t*-values. White labels indicate the coordinate of each slice in the MNI frame of reference (*x*, *z*).

For the contrast between resting and meditation states (RS > MS), the pgACC showed a reduced connectivity with the bilateral inferior parietal gyri but an increased connectivity with the MPFC, the left superior temporal gyrus (STG), and the right TPJ during MS as shown in Figure 2(a) and Table 3 (*p* < 0.05, FWE-corrected).

The dACC showed a decreased FC with the left anterior insula (AI) during MS (*p* < 0.05, FWE-corrected); see Figure 2(b) and Table 3.

The voxel-wise significance level was set at *p* < 0.001 with a spatial extent threshold of 16 contiguous voxels, yielding a whole brain threshold of *p* < 0.05 corrected for multiple comparisons using AlphaSim algorithm implemented in AFNI (data dimension: 61 × 73 × 61 voxels, Gaussian filter widths: FWHM*x* = 6.66, FHWM*y* = 6.88, and FWHM*z* = 6.79).

#### 3.2.2. Longitudinal Analysis of RSFC Changes due to Meditation

**Figure 3:**
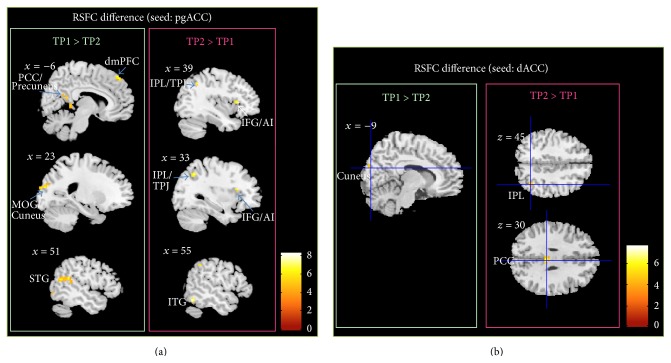
(a) Longitudinal seed-based RSFC results (seed = pgACC) (*p* < 0.05, FWE-corrected). Bar plot represents the *t*-values. White labels indicate the coordinate of each slice in the MNI frame of reference (*x*, *z*). (b) Longitudinal seed-based RSFC results (seed = dACC) (*p* < 0.05, FWE-corrected). Bar plot represents the *t*-values. White labels indicate the coordinate of each slice in the MNI frame of reference (*x*, *z*).

The longitudinal effect of meditation practice (TP1 > TP2) on RSFC was tested separately for pgACC and dACC. For pgACC, a decreased connectivity to the left PCC/precuneus, the left dorsal medial prefrontal cortex (dmPFC), the right superior temporal gyrus (STG), the left middle occipital gyrus, and left inferior temporal gyrus was observed, while connectivity to the right inferior temporal gyrus, the right inferior frontal gyrus (IFG), and the right TPJ/IPL increased after meditation training (*p* < 0.05, FWE-corrected; see Figure 3(a) and Table 4).

In dACC, meditation training entailed a reduction of RSFC to calcarine sulcus and the cuneus but an increased RSFC towards cerebellum, the right inferior parietal lobe (IPL), and the posterior cingulated cortex (PCC) (*p* < 0.05, FWE-corrected; see Figure 3(b) and Table 4).

The voxel-wise significance level was set at *p* < 0.001 with a spatial extent threshold of 16 contiguous voxels, yielding a whole brain threshold of *p* < 0.05 corrected for multiple comparisons using AlphaSim algorithm implemented in AFNI (data dimension: 61 × 73 × 61 voxels, Gaussian filter widths: FWHM*x* = 6.66, FHWM*y* = 6.88, and FWHM*z* = 6.79).

### 3.3. Group Independent Component Analysis

#### 3.3.1. Comparison of the DMN Independent Component between RS and MS

**Figure 4:**
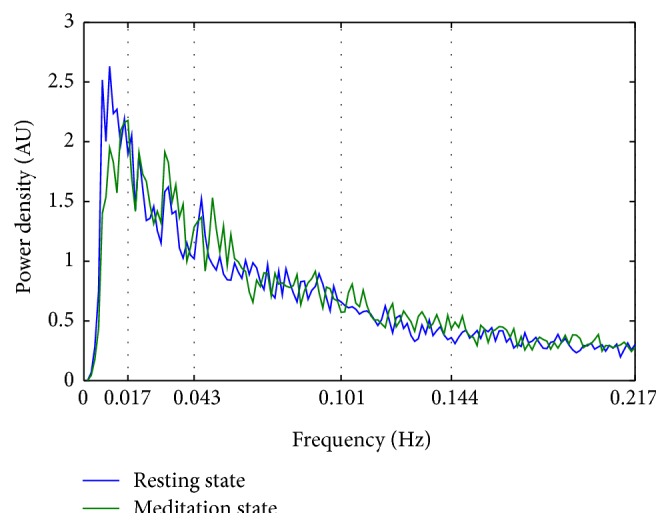
Mean power spectral density of the Default Mode Network (DMN) component. The peak power density was observed below 0.04 Hz for both conditions (resting state and meditation state). AU stands for arbitrary unit.

To ensure that our DMN comparison between these two conditions was performed in the same low frequency band, power density spectra were obtained. The results revealed that both DMNs were derived from the same very low frequency band (<0.04 Hz). This insured that the comparison between RS and MS were performed in the same “expected” very low frequency domain [60]; see Figure 4.

**Figure 5:**
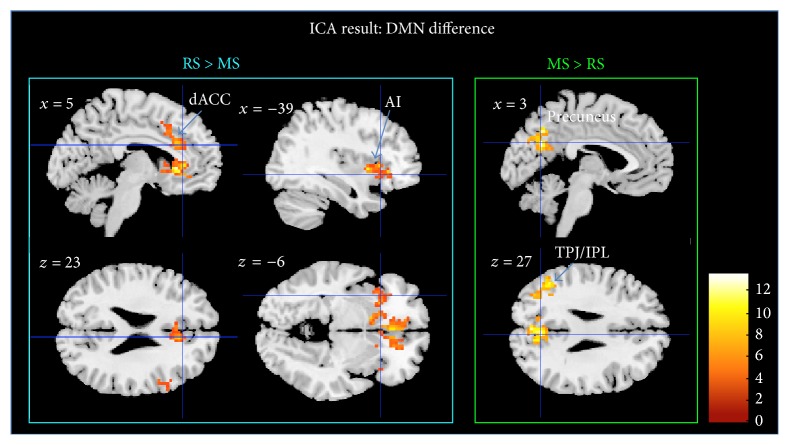
ICA results. DMN differences at TP2 for RS > MS and MS > RS (*p* < 0.05, FWE-corrected). Bar plot represents the *t*-values. White labels indicate the coordinate of each slice in the MNI frame of reference (*x*, *z*).

At TP2, ICA revealed reduced activation during meditation in dACC, sgACC, bilateral insula, superior frontal gyrus, and inferior frontal gyrus (IFG) when compared to rest (*p* < 0.05, FWE-corrected). The precuneus and the left temporoparietal junction (TPJ) showed an increased internal consistency in the ICs during MS; see Figure 5 and Table 3.

The voxel-wise significance level was set at *p* < 0.001 with a spatial extent threshold of 16 contiguous voxels, yielding a whole brain threshold of *p* < 0.05 corrected for multiple comparisons using AlphaSim algorithm implemented in AFNI (data dimension: 61 × 73 × 61 voxels, Gaussian filter widths: FWHM*x* = 7.39, FHWM*y* = 8.18, and FWHM*z* = 8.19).

#### 3.3.2. Longitudinal Analysis of RS Changes in the DMN Independent Component due to Meditation

The paired* t*-test at a threshold of *p* < 0.05, FWE-corrected, did not reveal longitudinal changes within the DMN independent components. The voxel-wise significance level was set at *p* < 0.001 with a spatial extent threshold of 23 contiguous voxels, yielding a whole brain threshold of *p* < 0.05 corrected for multiple comparisons using AlphaSim algorithm implemented in AFNI (data dimension: 61 × 73 × 61 voxels, Gaussian filter widths: FWHM*x* = 7.84, FHWM*y* = 8.68, and FWHM*z* = 8.53).

### 3.4. Regional Homogeneity (ReHo) Analysis

#### 3.4.1. ReHo Comparison between MS and RS

**Figure 6:**
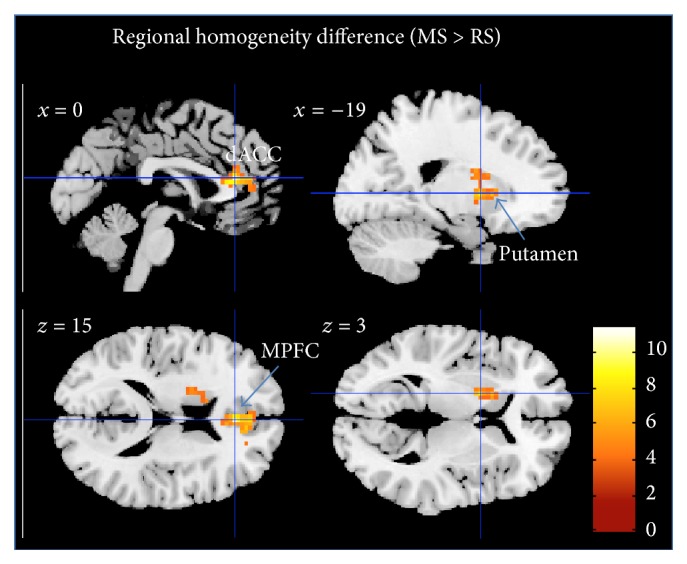
Regional homogeneity difference between MS and RS (*p* < 0.05, FWE-corrected). Bar plot represents the *t*-values. White labels indicate the coordinate of each slice in the MNI frame of reference (*x*, *z*).

The comparison of MS and RS (MS > RS) showed an increased ReHo in dACC, left striatum (putamen), MPFC, and TPJ (supramarginal gyrus), FWE-corrected at *p* < 0.05; see Figure 6 and Table 3.

The voxel-wise significance level was set at *p* < 0.001 with a spatial extent threshold of 63 contiguous voxels, yielding a whole brain threshold of *p* < 0.05 corrected for multiple comparisons using AlphaSim algorithm implemented in AFNI (data dimension: 61 × 73 × 61 voxels, Gaussian filter widths: FWHM*x* = 13.25, FHWM*y* = 13.98, and FWHM*z* = 12.13).

#### 3.4.2. Longitudinal Analysis of ReHo Changes

Longitudinal effects of meditation on the ReHo of RS activity were tested between time points using a paired* t*-test. However, no significant differences between time points were found. The voxel-wise significance level for this analysis was set at *p* < 0.001 with a spatial extent threshold of 70 contiguous voxels, yielding a whole brain threshold of *p* < 0.05 corrected for multiple comparisons using AlphaSim algorithm implemented in AFNI (data dimension: 61 × 73 × 61 voxels, Gaussian filter widths: FWHM*x* = 14.28, FHWM*y* = 14.53, and FWHM*z* = 12.48).

## 4. Discussion

### 4.1. Summary

In the current study, for the first time, we investigated longitudinal effects of mindfulness meditation training on changes in functional connectivity between brain areas. To our knowledge, no previous study has directly compared a meditation condition with a resting condition using sophisticated methods like ICA, ReHo, and FC. During the analyses, we specifically focused on target regions and resting state network playing a role in affective disorders like depression such as the pgACC, the dACC, and the DMN.

We found significant differences in functional connectivity both between states (rest versus meditation) and between time points (before versus after training). The ICA analysis showed differences in the internal consistency in the precuneus and the temporoparietal junction, increased during meditation, while the internal consistency of frontal brain regions decreased. The dACC further revealed reduced connectivity with anterior insula during meditation. As an indication of plastic changes following mindfulness meditation, reduced RSFC between the pgACC and dorsal medical prefrontal cortex was observed after meditation training.

### 4.2. Detailed Findings, Previous Work, and Explanations

#### 4.2.1. Imaging Findings

ICA revealed that, during MS, in comparison to RS, the DMN component had stronger association of TPJ and precuneus, while activity in frontal, cingulate, and insular cortex was less associated with DMN. This may reflect the expected network dissociation as a function of cognitive task during meditation.

#### 4.2.2. Psychometry Findings

Our findings indicate that the short-term practice of mindfulness meditation leads to differences in CES-D score before and after training. Nearly 50% reduction in depressive symptoms after mindfulness meditation training is consistent with recent meta-analyses reported by [3, 64].

Short-term meditation training yielded lowered acute feelings of tension at the time of scanning in our study. Likewise, our results emulate previous reports on the effects of meditation on well-being in anxiety trait and depression self-reports [65].

#### 4.2.3. Implication for Acute Effects and Plasticity Induction during Supporting Therapeutic Efficacy of Meditation

The reported beneficial effects of mindfulness meditation for the treatment of emotional dysregulation in major depression and other affective disorders led us to hypothesize longitudinal changes in the RSFC. Changes in self-reports suggest that meditation training are very well in accordance with enduring changes in brain function as found in the literature and also in our study [66, 67]. In this sense, the longitudinal observation of RSFC decreases is very well in line with cross-sectional observations of altered functional responses [25] and RS connectivity, especially in the DMN. As one key region mediating depression-related symptoms, pgACC showed a longitudinal reduction in its connectivity with the PCC/precuneus region and conversely increased its connectivity with the right IPL. Such a reduction of intrinsic connectivity between pgACC and the posterior DMN components in precuneus would indeed reflect the expected directionality of connectivity changes if a hyperconnectivity within DMN in major depressive disorder (MDD), being considered a target of the antidepressant efficacy of meditation training. Sheline and colleagues [20] reported that such hyperconnectivity in the DMN exists for MDD; and, in their study, they highlighted the importance of abnormal hyperconnectivity of a “dorsal nexus.” Quite consistently, we also found a reduction of connectivity between pgACC and dmPFC, close to the previously reported dorsal nexus region. That such changes of RSFC after interventions of antidepressant action can also be observed in healthy subjects was recently supported by a finding from Scheidegger and colleagues [68], who showed that, similar to our finding, a connectivity decrease of anterior and posterior DMN components, located in pgACC and PCC, was observed after 24 hrs of ketamine injection, mirroring the substances' maximum antidepressant effects in patients. In other words, the neuronal effects observed after 40 days of meditation, which was shown to affect depression-related psychometry, also reduced connectivity in a network that is hyperconnected in MDD, and, given the predominance of pgACC findings, this further suggests some specificity given the overall role of its dysfunction and abnormal connectivity in depression [29].

Longitudinal meditation training effects on functional connectivity are expected to be subserved by acute meditation effects when compared to resting conditions. Similar to the observed longitudinal reduction of RSFC between anterior and posterior DMN components we found an opposite pattern of connectivity changes from rest for pgACC compared to lateral and medial parietal DMN subcomponents. At the same time, the frontal DMN subcomponents seemed to aggregate as indicated by the increased RSFC between pgACC and dmPFC during meditation. Next to within-network connectivities our results showed convergence of state (between conditions) and trait (between time points) effects on the alteration of the functional connectivity between pgACC and right TPJ. Here increased connectivities between these two regions would be in line with functional role of TPJ in reorienting of attention [69]. In the meditation technique applied here, both focused attention and open monitoring aspects were combined. While increased pgACC-TPJ connectivity very well fits together with an improvement of open monitoring behavior such as “present moment awareness,” it would equally well fit into potential antidepressant efficacy reversing narrowed or biased attention [30], although we did not include a direct measure of such behavioral effects.

When investigating the effects on the attention maintenance network [27], dACC was found to exert a reduced connectivity with its functional counterpart in the AI in MS compared to RS (Figure 2(b)). Previous studies reported insula activity during meditation [12] and anatomical changes in terms of increased gray matter [70, 71]. The dACC region was however not found to show increased gray matter in either study. This structural distinction of subregions with and without volumetric differences within the cingulo-opercular network would be in line with our structural decoupling during meditation. In the literature there are also findings of increases in dACC activity, especially in expert meditators [72] and recent investigations of focused breathing versus mind wandering reported increased activation in both dACC and AI [36]. When interpreting our findings in line with functional involvements of dACC and AI, next to specifications of the explicit study design, one also has to acknowledge that one region can be part of several networks. This is especially true for AI, which next to its involvement in focused attention, together with dACC, is activated together with MPFC during self-referential conditions and further plays an important role in orchestrating different networks [73]. In contrast to the observed decoupling of AI and dACC in our seed-based analysis, the finding of reduced correlation with the DMN independent components of these two regions appears within a general observation of reduced IC connectivity in all task-positive regions (Figure 5). Here dACC and AI both showed similar effects of functional decoupling with DMN as an effect of differentiated task behavior during meditation, which however does not imply that they were necessarily any more functionally coupled. The differential effects on dACC and AI are also supported by the local metrics of the ReHo analysis.

Here, the comparison between RS and MS conditions revealed increased ReHo during MS in the MPFC and the dACC and subcortical regions but not in insula cortices. Since this is the first paper to our knowledge to apply ReHo measures to resting or active states in meditators, we cannot directly relate our observations to other findings. When trying to relate changes of temporal synchrony [62] to cross-sectional changes in patient populations, one likewise has to acknowledge that for MPFC both increases and decreases have been reported. While decreases have been for MPFC and ACC in depression [74], but also social anxiety [75] or Alzheimer disease [76], a recent study in bipolar depression rather indicated an increase in MPFC ReHo [74]. If indeed the functional distinction in increased versus decreased ReHo in bipolar versus MDD patients would mirror functional states that also discern rest versus active meditation, this would need to be subject to future investigations targeting the specificity and the physiological meaning of this observation. One important addition to the other connectivity-based findings is however provided by altered ReHo in putamen. This structure has been repeatedly reported in meditation studies [14, 77–80] but, distinct from the other main regions frequently reported, was not revealed by our connectivity analyses.

### 4.3. Strengths and Limitations of the Current Study

Mindfulness meditation and its neural correlates have been investigated in several studies [36, 81] by means of block-design fMRI. A limitation of such a design is that meditation is difficult to perform with a short on-and-off period (30–45 sec), especially for novice meditators. This limitation also results from the GLM itself, in which a longer epoch will decrease the design efficiency. However, the study of Bærentsen et al., 2010, investigated continuous meditation by means of fMRI. They performed both SPM GLM and ICA on continuous meditation and focused on the difference between the meditation onset period and the maintained state of meditation.

One may argue that independent component comparison still would best be performed for components identified across all conditions. In our case, however, similar to Harrison et al., 2008, who used a continuous sad mood induction paradigm compared to RS, we decided to decompose independent components for both conditions separately, also following the method of Calhoun et al., 2008, who used ICASSO to insure ICA stability.

As we did not recruit a control group, our findings cannot be causally attributed solely to mindfulness meditation so this will be important improvement for future directions of further investigation.

Moreover, the small sample size, due to discarding of incomplete subjects and imaging artifacts due to motion inside the scanner, limits the generalizability of findings. An increased sample size will increase the statistical power in a future study.

At baseline, the CES-D score of our sample slightly surpassed the clinical cutoff for depression (CES-D > 16), which could be a potential selection bias. Despite a negative diagnosis of depression based on a personal interview, some of the participants may have had a subclinical depression. In this aspect, our sample may still be representative of the general population due to the high prevalence of subclinical depression in general population; however inference to entirely healthy population is limited.

In our present study we did not collect physiological data. Future studies might consider inclusion of certain parameters, such as heart rate, breath rate, and respiration amplitude. High-frequency heart rate variability (HF-HRV) which is a measure of parasympathetic nervous system output that has been associated with enhanced self-regulation [80] would be of particular interest. Since meditation has been shown to increase HF-HRV, this might serve as a biomarker for meditation training-related effects. Recent findings however also suggest a direct relationship especially between HF-HRV and RSFC of, for example, cingulate regions [82], so that functional interpretation of connectivity changes could thus be directly related to autonomous nervous system tone. Further studies on MDD and other affective disorders may help to clarify the actual role of these changes in emotional regulation after short-term meditation, as well as its therapeutic effects.

## 5. Conclusion

We were able to show that the 40-day mindfulness meditation training resulted not only in amelioration of depression-related symptoms but also in changes in whole brain networks towards connectivity states usually found when comparing healthy controls to MDD patients in clinical studies. These longitudinal changes were in part mirrored in short-term effects when directly comparing RS and MS. This was especially evident in the involvement of the temporoparietal junction and its connectivity to anterior cingulate cortex, demonstrating cross-network interaction. Within-network consistency was most strongly affected in the DMN where anterior and posterior subcomponents segregated both longitudinally and in direct comparison of MS and RS. In particular effects in dACC which also showed altered local fluctuations during MS and in consequence strongest decoupling from DMN in direct comparison to RS spatially overlap with a network related to attention maintenance [27]; however individual analysis steps revealed variable cluster locations within anatomical boundaries of the midcingulate cortex. These network effects were in part paralleled by observations of altered local fluctuations, which further supported distinct effects in subregions of the salience network, as also supported by reduced connectivity between insula and dACC. Finally we could again identify the putamen as an important subcortical region during meditation, characterized by increased local synchronization of signal variations. Critically, our results may provide insight into the brain circuits that potentially subserve the plastic antidepressant effects of mindfulness meditation training.

## Figures and Tables

**Table 1:** Characteristics of the subject sample. Wherever relevant, group mean (± standard deviation) is given.

Number of participants	13
Ratio of males and females	3/10
Age	24.53 (±5.89)
Ratio of handedness	right = 13, left = 0

Minutes of meditation practice per day	10.67 (±1.66)
Total number of meditation days until scanning	39.23 (±3.63)
Total number of minutes of meditation practice until scanning	418.07 (±71.45)

**Table 2:** Profile of Mood States (POMS), Center for Epidemiologic Studies Depression Scale (CES-D), and State-Trait Anxiety Inventory (STAI) scores before and after meditation training. Group means and standard deviations are listed. Significant group differences are listed as follows: *∗∗∗*: significant at *p* < 0.001, *∗∗*: significant at *p* < 0.01, and *∗*: significant at *p* < 0.05. *※*: before training, 6 out of 13 subjects surpassed the cutoff for depression. (+): positive mood state factor, (−): negative mood state factor.

Questionnaire	Before training	After training
POMS		
Anger (−)	2.84 (±3.78)	3.30 (±6.07)
Fatigue (−)	4.84 (±2.67)	3.69 (±4.57)
Vigor (+)	11.92 (±5.31)	12.30 (±5.36)
Friendliness (+)	18.92 (±2.98)	19.46 (±4.27)
Tension (−)	8.00 (±3.36)	5.38 (±5.18)^*∗*^
Depression (−)	3.76 (±3.94)	4.53 (±8.04)

CES-D	16.23 (±9.54)^*※*^	9 (±6.20)^*∗∗∗*^

STAI, state	17.84 (±4.75)	14.38 (±10.16)
STAI, trait	21.30 (±8.6)	16.84 (±9.56)^*∗∗*^

**Table 3:** Regions showing functional differences between conditions (rest versus meditation).

Analysis	Rest > meditation					Meditation > rest				
	Region	Side	*K*	MNI coordinates	*z*-score	Region	Side	*K*	MNI coordinates	*z*-score
ICA	(*p* < 0.001, FWE^a^-corrected cluster threshold at *p* < 0.05)^b^									
	dACC	R	202	9 21 36	5.70	Precuneus	R	178	3 −66 27	4.94
	sgACC	R	349	9 27 0	5.60	TPJ/IPL	L	157	−51 −57 27	4.31
	AI	L	336	−39 9 0	5.02					
	AI	R	37	45 18 0	4.34					
	IFG/BA9	R	21	60 18 27	4.26					

Seed-based FC	(*p* < 0.001, FWE-corrected cluster threshold at *p* < 0.05)^b^									
(ROI: pgACC)	IPL	L	42	−45 −39 36	4.31	TPJ	R	58	57 −66 36	4.51
	IPL	R	45	39 −51 51	4.17	MPFC	R	36	9 51 9	4.49
						STG	L	23	−42 −60 18	3.86
(ROI: dACC)	AI	L	20	−36 18 3	4.2	n.s.				

ReHo	(*p* < 0.001, FWE-corrected cluster threshold at *p* < 0.05)^b^									
	n.s.					dACC	R	125	0 36 15	5.32
						MPFC	R	125	6 48 12	4.24
						Putamen	L	77	−21 6 3	4.40

Notes: AI, anterior insula; dACC, dorsal anterior cingulate cortex; IPL, inferior parietal lobe; K, cluster size; MPFC, medial prefrontal cortex; pgACC, pregenual anterior cingulate cortex; sgACC, subgenual anterior cingulate cortex; STG, superior temporal gyrus; TPJ, temporoparietal junction; and BA: brodmann area.

^a^Family-wise error.

^b^A combined threshold of *p* < 0.001 and a minimum cluster size determined by AlphaSim^c^ algorithm in AFNI, resulting in FWE-corrected threshold of *p* < 0.05. An estimate of the spatial correlation across voxels was modeled using the program 3dFWHM^d^ in AFNI.

^c^AlphaSim: http://afni.nimh.nih.gov/pub/dist/doc/manual/AlphaSim.pdf.

^d^3dFWHM: http://afni.nimh.nih.gov/pub/dist/doc/program_help/3dFWHM.html.

**Table 4:** Regions showing RSFC differences between time points.

Analysis	TP1 > TP2					TP2 > TP1				
	Region	Side	*K*	MNI coordinates	*z*-score	Region	Side	*K*	MNI coordinates	*z*-score
ICA	n.s. (*p* < 0.001, FWE^a^-corrected cluster threshold at *p* < 0.05)^b^									

Seed-based FC	(*p* < 0.001, FWE-corrected cluster threshold at *p* < 0.05)^b^									
(ROI: pgACC)	PCC/precuneus	L	58	−12 −51 15	4.71	ITG	R	16	54 −63 −18	4.23
	dmPFC	L	49	−12 45 54	4.35	IFG	R	17	39 24 6	3.84
	STG	R	53	54 −63 15	4.34	IPL/TPJ	R	18	33 −60 42	3.76
	MOG	L	22	−24 −102 6	4.19					
	ITG	L	21	−57 0 −24	4.01					
(ROI: dACC)	Calcarine/cuneus	L	18	−9 −84 21	4.37	Cerebellum	L	22	−30 −54 −30	4.56
	BA19	L	26	−27 −90 21	4.01	IPL	R	16	36 −60 45	4.20
						PCC	R	19	$\begin{array}{ccc} 3 & -30 & 30 \end{array}$	4.13

ReHo	n.s. (*p* < 0.001, FWE-corrected cluster threshold at *p* < 0.05)^b^									

Note: dmPFC, dorsal medial prefrontal cortex; IFG, inferior frontal gyrus; IPL, inferior parietal lobe; ITG, inferior temporal gyrus; *K*, cluster size; PCC, post cingulate cortex; STG, superior temporal gyrus; MOG, middle occipital gyrus; and BA: brodmann area.

^a^Family-wise error.

^b^A combined threshold of *p* < 0.001 and a minimum cluster size determined by AlphaSim^c^ algorithm in AFNI, resulting in corrected threshold of *p* < 0.05. An estimate of the spatial correlation across voxels was modeled using the program 3dFWHM^d^ in AFNI.

^c^AlphaSim: http://afni.nimh.nih.gov/pub/dist/doc/manual/AlphaSim.pdf.

^d^3dFWHM: http://afni.nimh.nih.gov/pub/dist/doc/program_help/3dFWHM.html.
